# A smartphone image dataset with lux-meter ground truth for workplace illuminance estimation

**DOI:** 10.1016/j.dib.2026.113134

**Published:** 2026-08-03

**Authors:** Anton Buryi

**Affiliations:** Department of Environmental and Occupational Health, California State University, 18111 Nordhoff Street, Northridge, CA, 91330-8412, United States

**Keywords:** Occupational exposure assessment, Computer vision, Machine learning, Lighting measurement, Indoor environment, Image annotation, Photometric monitoring, Occupational health

## Abstract

A controlled image dataset of 5814 smartphone photographs is presented for training and benchmarking machine-learning models for indoor illuminance (lux) estimation. All images were acquired using a Samsung Galaxy A16 smartphone mounted on a tripod under fixed camera parameters such as ISO 50, exposure time 1/50 s, white balance 4000 K, 45-degree camera tilt, and 31 cm camera height above the surface. Ground-truth illuminance was recorded using a Lutron LX-101A lux meter at one central point (2300 images) or at five fixed spatial points per image (3514 images). The dataset spans 10 distinct table surfaces and 22 colored paper types photographed across different illuminance levels generated by adjustable LED lamps with correlated color-temperature settings. Surface categories include colored paper (2376 images), white paper (1563 images), real table surfaces (1505 images), and other surfaces (370 images). The dataset enables training and testing of machine learning models for illuminance estimation at defined points on a surface and supports assessment of color classifier performance under varying illuminance conditions. The dataset is publicly deposited in the Zenodo repository (https://doi.org/10.5281/zenodo.20499311).

Specifications Table

**Table:** 

Subject	Health Sciences, Medical Sciences & Pharmacology
Specific subject area	Smartphone image dataset for workplace illuminance estimation using machine learning
Type of data	Image; Table; Figure; Graph Raw; Analyzed; Processed
Data collection	Images were acquired with a Samsung Galaxy A16 smartphone mounted on a tripod under fixed manual settings: ISO 50, 1/50 s exposure, 4000 K white balance, 45° tilt, and 31 cm height. Ground-truth illuminance was measured using a Lutron LX-101A digital lux meter at one central point or five fixed spatial points per image. The lux-meter sensor was removed from the field of view before each photograph was captured.
Data source location	Los Angeles County, California, USA. Primary data collection locations included California State University, Northridge, Northridge, California, and Marina del Rey, California. Additional images were collected at indoor locations in the Los Angeles area under the same controlled camera and measurement protocol.
Data accessibility	Repository name: Zenodo Data identification number: 10.5281/zenodo.20499311 (dataset, all versions); 10.5281/zenodo.20658590 (analysis code) Direct URL to data:https://doi.org/10.5281/zenodo.20499311; https://doi.org/10.5281/zenodo.20658590 **Instructions for accessing these data:** The complete image dataset, master annotation CSV (annotation_master_latest.csv; 5814 rows × 23 columns), full-dataset feature table (feature_master_latest.csv; 5814 rows × 133 columns), machine-readable data dictionaries, illuminance-distribution statistics, measurement-point (region of interest, ROI) pixel coordinates, and train–test split files are publicly available at the Zenodo dataset record above. All files, including the complete image ZIP archives, are contained in the current version of the Zenodo dataset record; the concept DOI above always resolves to the latest version. All reported analyses used the annotation and feature tables introduced in version 3 (10.5281/zenodo.20657011), which are unchanged in the current version. Analysis code and feature-extraction workflows are available at https://doi.org/10.5281/zenodo.20658590.
Related research article	None

## 1 Value of the Data

• To the author’s knowledge, this is the first publicly available smartphone image dataset with simultaneous lux-meter ground truth recorded at up to five spatial positions per image, enabling controlled-condition benchmarking of image-based illuminance estimation. Existing computer-vision lighting datasets, such as those for intrinsic image decomposition, record only relative shading and contain no absolute photometric ground truth in physical units.
• The five-point annotations (3514 five-point images) enable a distinctive reuse scenario absent from prior datasets: the study of spatial illuminance gradients across a single surface, including gradient-aware model designs and uniformity assessment relevant to workplace lighting standards.
• The 22 colored-paper types photographed across approximately 50–1600 lx enable research on illuminance-dependent color appearance and color classification under controlled dimming, relevant to color-critical occupational tasks.
• Grouping variables (session, physical measurement point) and deposited train–test split files support leakage-free machine-learning benchmarking and exact reproduction of the example analyses reported here.
• Potential user communities include occupational health and industrial hygiene researchers, indoor environmental quality researchers, computer vision researchers, lighting engineers, and developers of low-cost screening tools.
• The dataset reflects a controlled acquisition design: a single smartphone model, fixed manual camera settin gs, fixed viewpoint, and indoor night-time conditions. It is intended for benchmarking and method development and complements, rather than replaces, professional lux-meter measurement in occupational and indoor environmental assessment.

## 2 Background

Adequate indoor illuminance is an important occupational and indoor environmental health requirement. Workplace illuminance is commonly assessed using professional lux meters, which remain the reference method for direct measurement [[1], [2], [3], [4]]. However, manual assessment requires trained personnel, defined measurement positions, and careful placement of the sensor. When several points must be measured on the same work surface, differences in point selection and sensor placement can reduce reproducibility between operators or repeated assessment cycles.

Computer vision and machine-learning methods may support future tools for more standardized image-based screening of workplace illuminance. Previous studies have investigated image-based or machine-learning-based indoor illuminance estimation [5,6], but publicly available controlled smartphone image datasets with lux-meter ground truth remain limited. In particular, existing computer-vision lighting datasets, such as those developed for intrinsic image decomposition, record only relative shading without absolute photometric ground truth in physical units, and previously reported image-based approaches typically require room-specific training data.

Development and comparison of such methods require publicly available image datasets with ground-truth lux measurements, fixed camera settings, documented geometry, and surface variation. This dataset was collected as a first step toward that goal: to provide a controlled, lux-meter-annotated smartphone image collection for testing illuminance estimation models.

## 3 Data Description

The dataset is deposited in Zenodo under a versioned dataset record. All results reported in this article were computed on version 3 of the dataset (version-specific DOI: 10.5281/zenodo.20657011, published June 12, 2026); the concept DOI 10.5281/zenodo.20499311 always resolves to the latest version. Earlier versions contained annotation-table errors arising from filename parsing: version 3 corrected the surface-category assignment of 494 rows and the annotation type (label_kind) of 935 rows, reclassified 467 white-paper images previously grouped as colored paper, and normalized malformed surface names; ground-truth lux values and image files were unchanged, as documented in the version notes on the Zenodo record. The current version of the record is self-contained: it includes the complete image ZIP archives together with all documentation files, and the annotation and feature tables are unchanged from version 3. The dataset record includes the raw image files, the master annotation table, the full-dataset feature table, machine-readable data dictionaries, illuminance-distribution statistics, ROI pixel coordinates, and train–test split files. The analysis-code record contains scripts and workflows used for feature extraction and example analyses; the repository README identifies the script and data files that generate each figure and table in this article.

Dataset repository: Zenodo, https://doi.org/10.5281/zenodo.20499311 [7]
Analysis code: https://doi.org/10.5281/zenodo.20658590 [8]
License: CC BY 4.0

The raw image dataset contains 5814 JPEG smartphone photographs. The master annotation file (annotation_master_latest.csv; 5814 rows × 23 columns) provides image-level metadata, surface identifiers, lux measurements, and annotation type. The derived feature table (feature_master_latest.csv; 5814 rows × 133 columns) contains image identifiers, metadata, lux labels, ROI coordinates, basic image-derived features, square-context features, and spatial-difference features. Machine-readable data dictionaries (data_dictionary_annotation.csv and data_dictionary_features.csv) define every column of both files, including definition, data type, unit, allowed values or range, and role in the dataset (ground-truth label, metadata, grouping variable, or derived image feature).

All lux measurements and surface identifiers are encoded directly into each image filename using the following structure:

F{session} __T{table}__LC{lux_C}_UL{lux_UL}_UR{lux_UR}_LR{lux_LR}_LL{lux_LL}__{surface}.jpg.

For images with only a central measurement, the four corner fields are omitted. Example:

F001 __Tbeige__LC250_UL239_UR220_LR248_LL240__blush_paper.jpg.

In this filename, F001 indicates session 1, Tbeige indicates the beige laminate table, LC250 indicates 250 lux at the center, UL/UR/LR/LL indicate corner lux values, and blush_paper indicates the surface type. The lux values encoded in filenames are duplicated by design in the annotation-table columns, so users may rely on either source for ground-truth labels.

### 3.1 Important note on data leakage

Ground-truth lux values are encoded in image filenames for traceability, and the annotation table stores them in the lux_C, lux_UL, lux_UR, lux_LR, lux_LL, central_lux, and target_lux columns. Filenames, filename-derived strings, session identifiers, and these lux columns are labels and grouping variables only and must never be used as model inputs; doing so constitutes severe target leakage. The deposited train–test split files should be used to reproduce the reported example analyses (Table 1).

**Table 1:** Dataset composition by surface category and annotation type.

Surface category	1-point images	5-point images	Total
Colored paper	946	1430	2376
White paper	677	886	1563
Real table surfaces	677	828	1505
Other surfaces	0	370	370
Total	2300	3514	5814

The “other” category (370 images, all five-point, illuminance 68–1050 lux) comprises: (i) printed surface-texture sheets — 18 distinct table/wood-texture patterns printed on paper (surface identifiers print01–print18, 348 images) — introduced to increase the diversity of table-like appearances beyond the physical tables available at the collection sites; (ii) 21 single colored-paper images in alternative (horizontal) sheet orientations acquired during orientation tests; and (iii) one auxiliary photograph documenting the measurement arrangement, which was not used in any example analysis and should be excluded from modeling subsets. Users who require homogeneous subsets should filter on surface_group_norm in {white_paper, table, colored_paper}.

The distribution of measured center-point illuminance is summarized in Fig. 5 and in the deposited file lux_distribution_stats.csv, which reports percentiles, means, and standard deviations overall and by surface category and annotation type. Center-point values span 2–2280 lx; including the corner measurement points, recorded values span 1–2280 lx. Representative dataset images across surface categories and illuminance levels are shown in Fig. 6.

### 3.2 Example reuse analyses

**Fig. 1:**
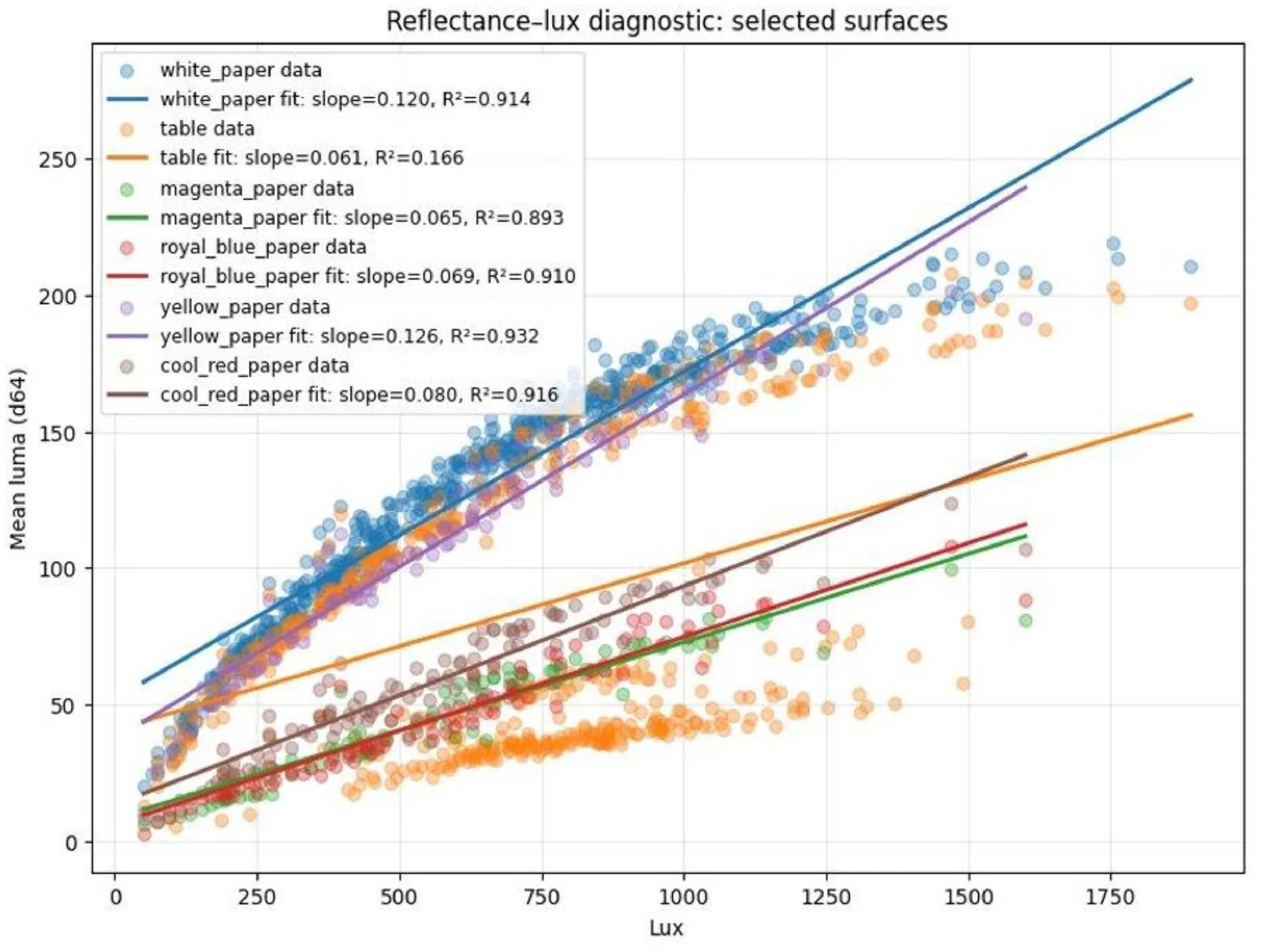
Mean image luminance from the central measurement point plotted against measured lux for selected surface types. Linear fits and coefficient-of-determination values are shown for descriptive comparison of the relationship between image luminance and reference lux measurements across surfaces.

**Fig. 2:**
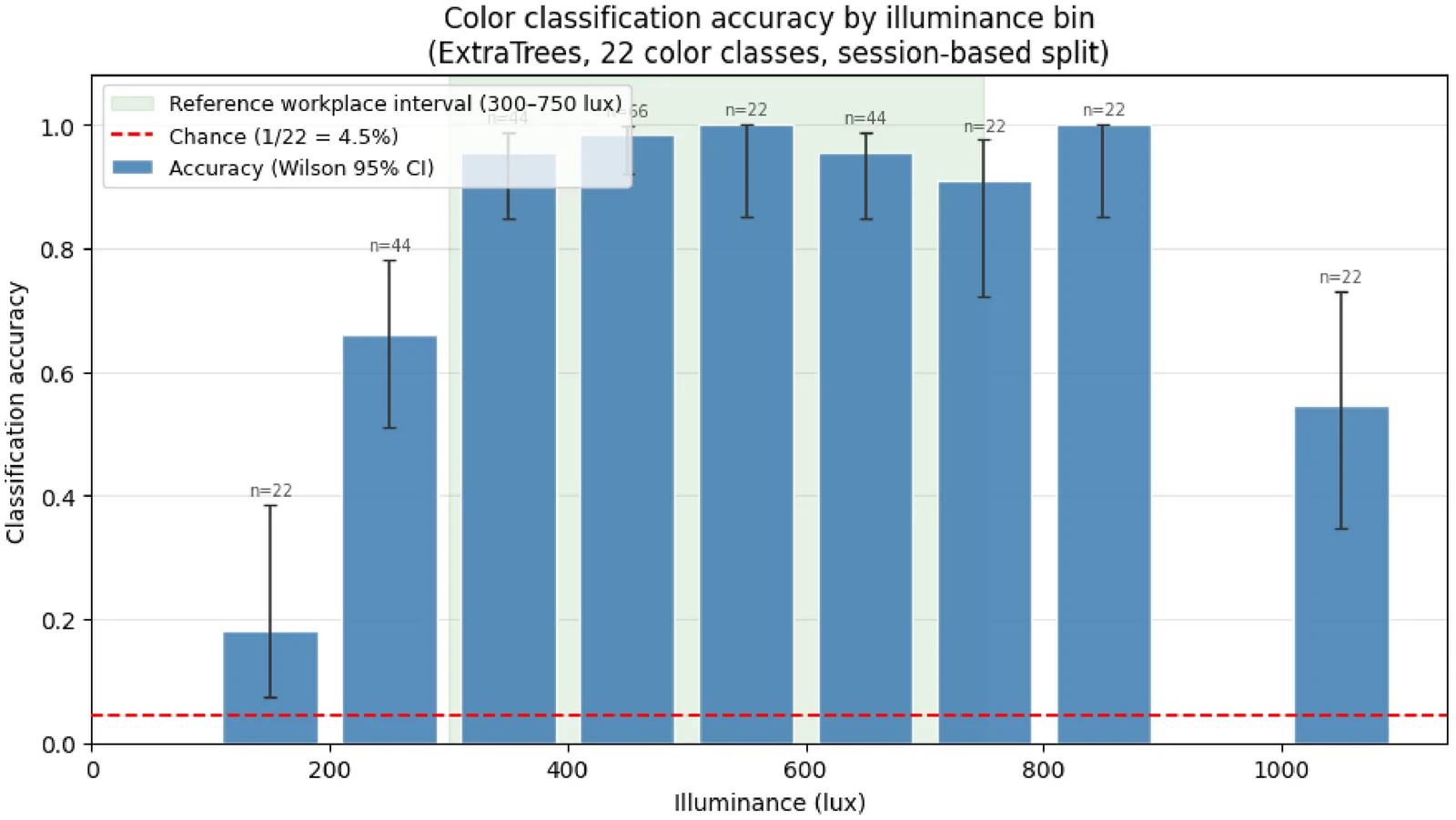
Color-classification accuracy by illuminance bin for the test set (ExtraTrees, 22 color classes, session-based grouped split; test n = 308, drawn from 1428 eligible five-point colored-paper images; training n = 1120). Dashed line indicates random chance (1/22 = 4.5%). The shaded region indicates a reference workplace-lighting interval of 300–750 lux. Error bars denote Wilson 95% confidence intervals; per-bin sample sizes are annotated above each point. The underlying figure-level data are deposited as Fig. 2_color_classification_bins_CI.csv.

**Fig. 3:**
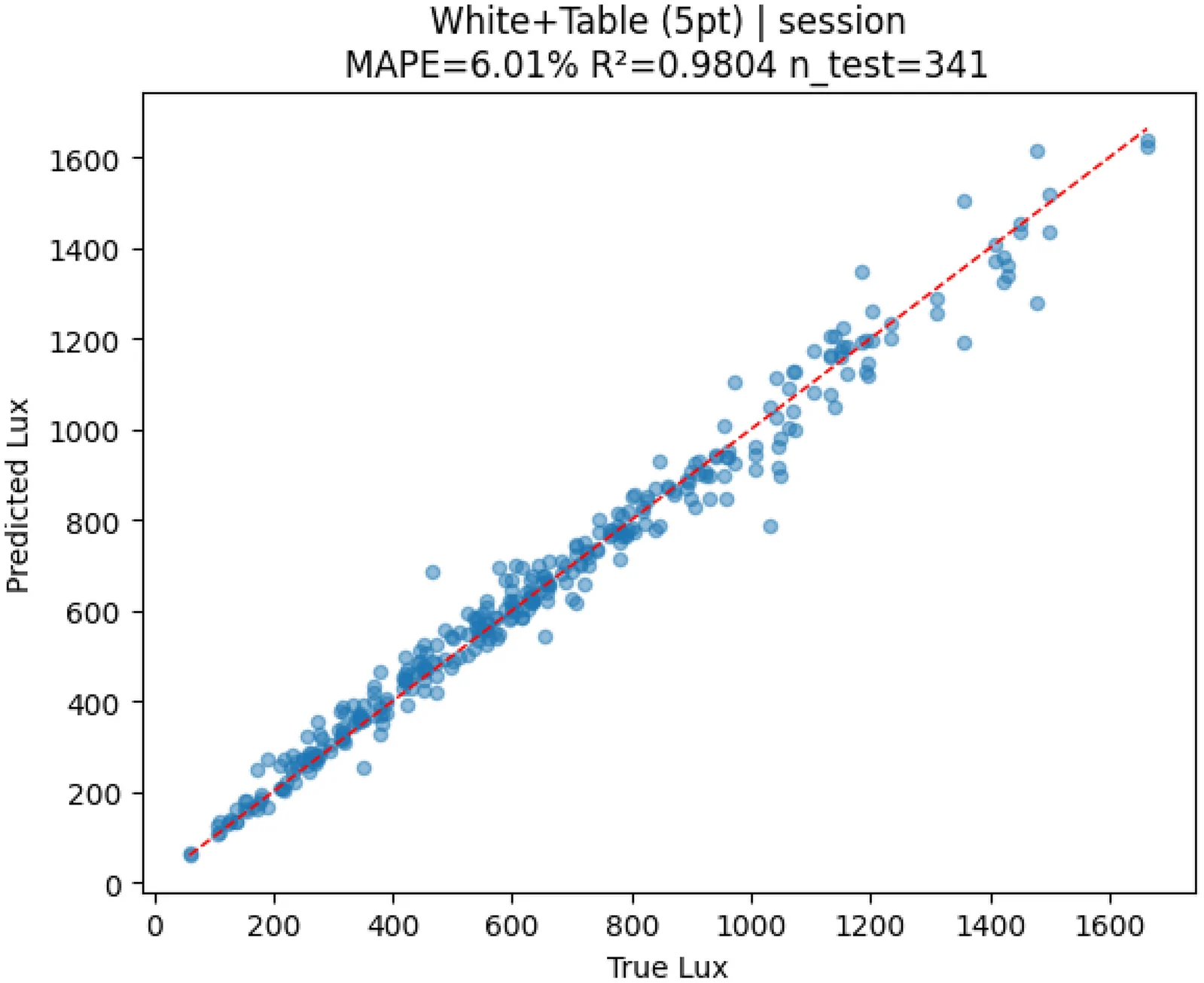
Predicted versus measured illuminance for the combined white-paper and table-surface subset using an ExtraTrees regression model, five-point annotation, and session-based grouped splitting. Training set: 1373 images; test set: 341 images; mean absolute percentage error (MAPE) = 6.01%; coefficient of determination (R^2^) = 0.980.

**Fig. 4:**
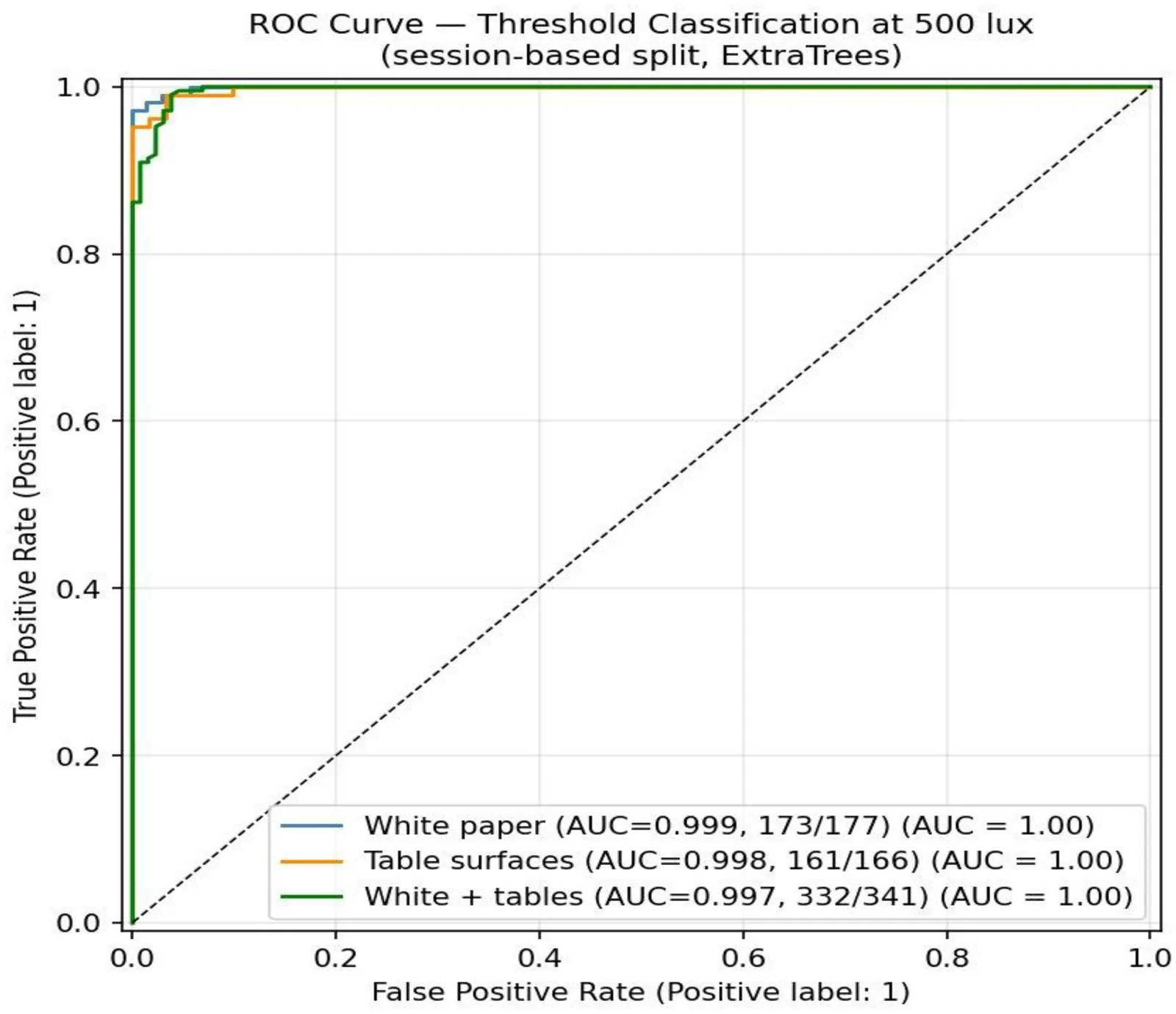
Receiver operating characteristic (ROC) curves for threshold-based illuminance classification at 500 lux across three surface subsets using ExtraTrees models, five-point annotation, and session-based grouped splitting. White paper: area under the curve (AUC) = 0.999, 173 of 177 test images correct. Table surfaces: area under the curve (AUC) = 0.998, 161 of 166 test images correct. White paper plus table surfaces: area under the curve (AUC) = 0.997, 332 of 341 test images correct.

Example analyses were performed to illustrate possible reuse of the dataset. Fig. 1 shows a diagnostic comparison between mean image luminance and measured lux for selected surface types. Fig. 2 shows an example color-classification analysis using the colored-paper subset, which may be relevant for studying image-based color recognition under varying illumination conditions [9]. Fig. 3 shows predicted versus measured lux values for an ExtraTrees regression model trained on the combined white-paper and table-surface subset. Fig. 4 shows receiver operating characteristic curves for threshold-based illuminance classification at 500 lux across three surface subsets. These analyses are provided as reproducible benchmark examples rather than as final validation of a deployment-ready illuminance-estimation system. The exact train–test split assignments used in Fig. 2, Fig. 3, Fig. 4 (scikit-learn GroupShuffleSplit, test_size = 0.2, random_state = 42, grouped by session) are deposited as CSV files (split_session_white_plus_table_seed42.csv, split_session_white_paper_seed42.csv, split_session_table_seed42.csv, and split_session_colored_paper_seed42.csv for the color-classification example, train = 1120, test = 308), together with figure-level data files (Fig. 1_luminance_lux_values.csv; Fig. 2_color_classification_bins_CI.csv with per-bin sample sizes and Wilson 95% confidence intervals). Two interpretive notes apply to these examples. First, although session-based splitting prevents images from the same acquisition session from appearing in both training and test sets, similar lighting configurations may recur across sessions conducted at the same locations with the same lamps; session-split metrics may therefore be optimistic relative to entirely novel environments, and the physical-point split provides a stricter estimate. Second, the weak luminance–lux relationship observed for bare tables (R² = 0.166, Fig. 1) reflects the confounding of surface reflectance with illuminance — dark and light tables at the same illuminance produce very different image brightness; this reflectance–illuminance ambiguity is a property the dataset is designed to expose and motivates the standardized white-paper protocol and future reflectance-invariant approaches.

**Fig. 5:**
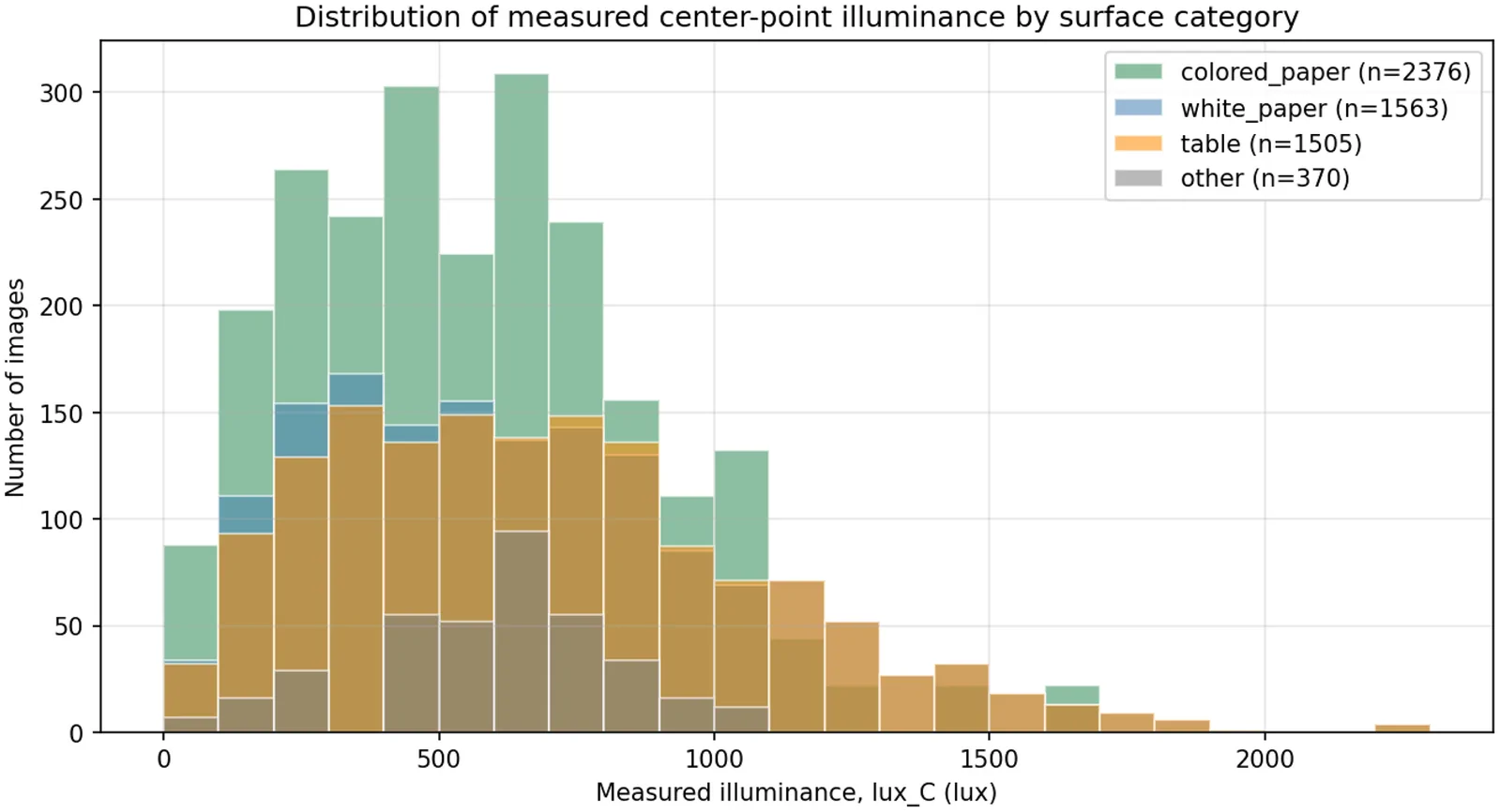
Distribution of measured center-point illuminance (lux_C) by surface category (100-lux bins).

**Fig. 6:**
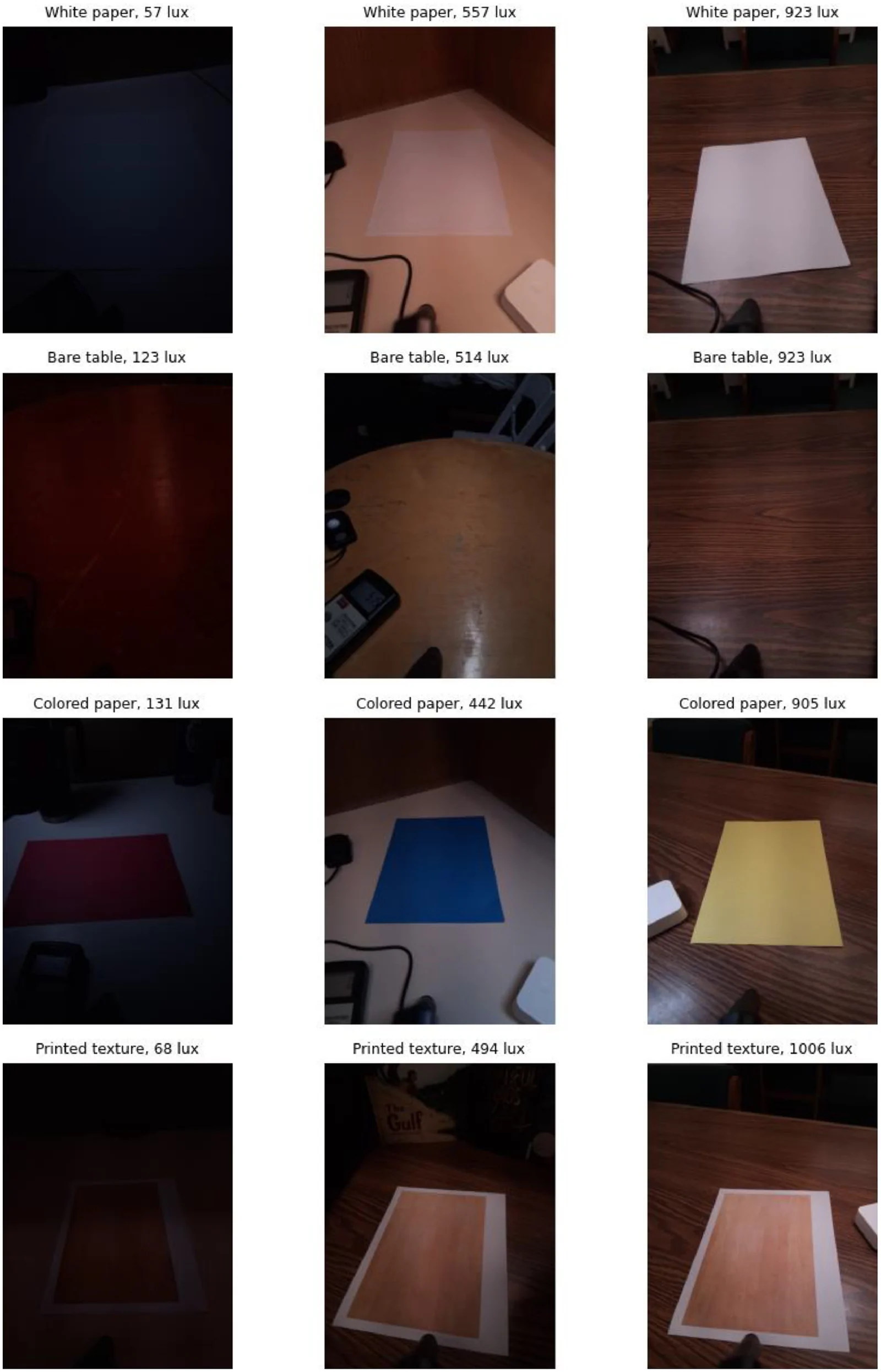
Representative dataset images across surface categories (white paper, bare table, colored paper, printed texture sheet) at low, medium, and high illuminance.

## 4 Experimental Design, Materials and Methods

### 4.1 Equipment and instrumentation

**Fig. 7:**
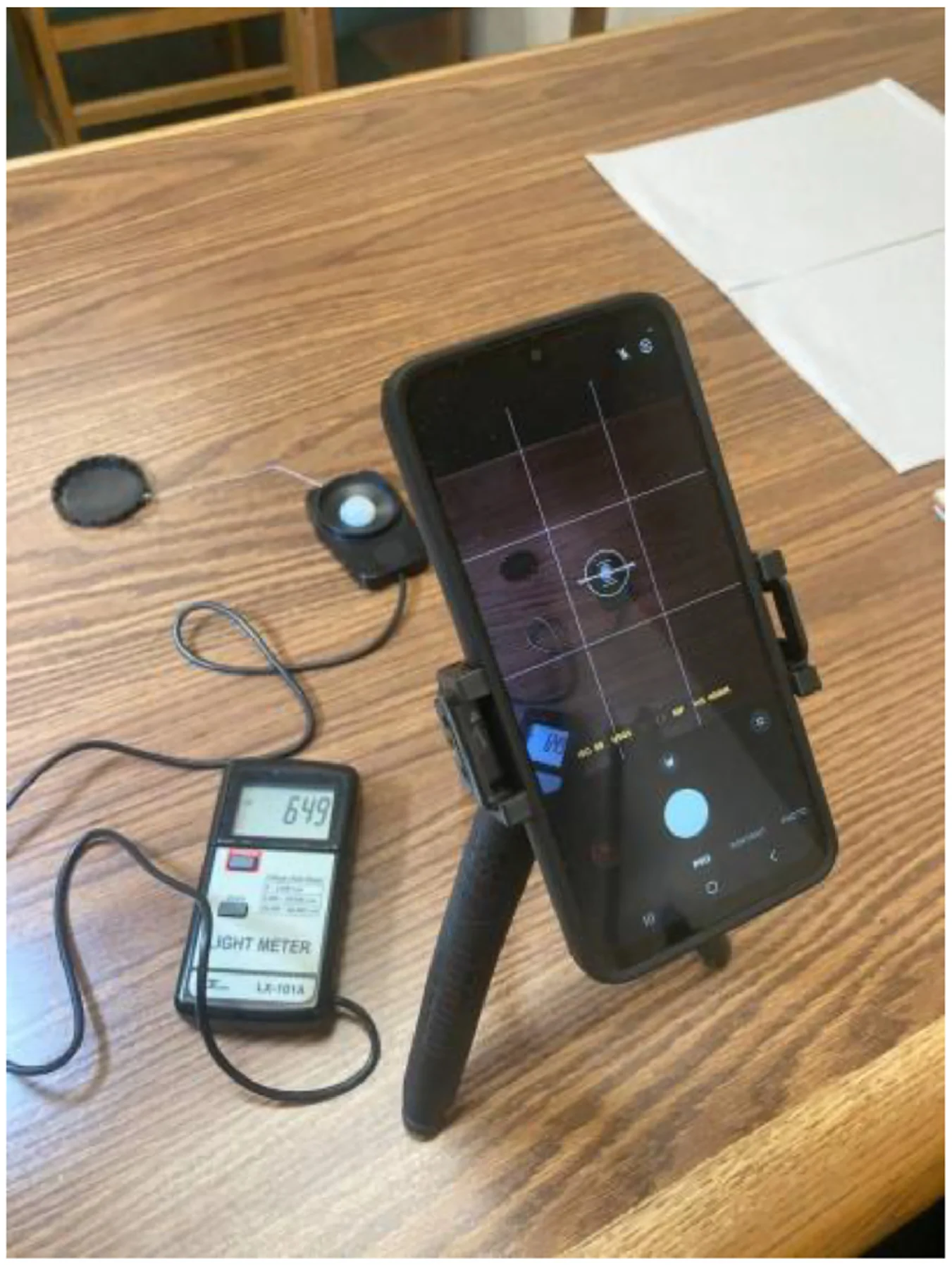
Data collection setup. The samsung galaxy A16 smartphone was mounted on a tripod in manual (PRO) mode with a grid overlay visible on the screen. The circle at the grid center and the four corner grid intersections defined the five fixed measurement locations. The Lutron LX-101A lux-meter sensor was positioned at each defined location during measurement and removed before image capture.

Photographs were collected using a Samsung Galaxy A16 smartphone mounted on a flexible tripod (UBeesize) in manual (PRO) mode. The tripod legs were locked in a fixed configuration for the duration of data collection; before every collection session, the 31 cm lens height was verified with a stainless-steel rule (Empire) and the 45° tilt was verified with a digital protractor (Husky), so positional consistency was actively confirmed rather than assumed. Camera settings were fixed across data collection sessions to improve imaging consistency: ISO 50, exposure time 1/50 s, white balance 4000 K, 45° camera tilt relative to the measured surface, and 31 cm camera height above the surface. Ground-truth illuminance was measured using a Lutron LX-101A digital lux meter (Lutron Electronic Enterprise Co., Ltd., Taiwan). On the 0–1999 lux measuring range, which covers 99.9% of the dataset (5806 of 5814 images), the meter has a display resolution of 1 lux and a manufacturer-rated accuracy of ±5% of reading; the remaining 8 images (2200–2280 lux) were measured on the 2000–19,990 lux range (resolution 10 lux, accuracy ±5% of reading), consistent with these values being multiples of 10 lux. The lux meter was purchased new specifically for this study and was supplied with a manufacturer’s inspection/calibration certificate (the manufacturer’s quality-management system is ISO 9001-certified by SGS); the certificate is provided with the dataset. No independent ISO/IEC 17025-accredited calibration or study-specific uncertainty evaluation was performed, so the manufacturer-rated accuracy is reported as an instrument specification rather than as a traceable measurement uncertainty. As with silicon-photodiode lux meters generally, cosine response and spectral response under LED illumination may deviate from ideal behavior, which is acknowledged as a source of ground-truth uncertainty common to this instrument class. The lux-meter sensor was always removed from the field of view before image capture and is not visible in any photograph in the dataset. The data collection setup is shown in Fig. 7.

### 4.2 Measurement and image-capture sequence

For one-point images, the lux-meter sensor was placed at the center measurement location, the reading was recorded by photographing the lux-meter display, the sensor was removed from the field of view, and the photograph was captured within approximately one second of sensor removal. After the sensor was placed at any measurement location, the displayed value was allowed to stabilize before recording: the reading was typically stable within approximately one second; when the display fluctuated, a few additional seconds were allowed until the value remained constant, and the settled value was recorded.

For five-point images, the sensor was placed sequentially at the center (C), upper-left (UL), upper-right (UR), lower-right (LR), and lower-left (LL) locations. Readings were likewise recorded by photographing the lux-meter display rather than writing values manually, which minimized total measurement time; in a timing check, the full five-point sequence required approximately 30 s. After the four peripheral measurements, the center point was re-measured immediately before image capture. No formal numerical rejection threshold was predetermined for this comparison; in practice, the photograph proceeded only when the two center readings agreed within approximately 1–3 lux on the meter’s 1-lx-resolution range (within one display count, i.e., 10 lux, for the eight acquisitions on the 10-lx-resolution range), and sequences showing greater variation were repeated immediately. The value recorded immediately before capture was used as the center ground-truth label. The sensor was then removed and the photograph captured within approximately one second. After image capture, the center point was measured once more; acquisitions whose post-capture reading showed greater variation than this working tolerance were deleted and re-acquired. The number of repeated acquisitions was not logged; no complete deposited sessions were subsequently excluded because of drift. During approximately the first 20 sessions, all five locations were re-checked after image capture to confirm the adequacy of the center-only check; in later sessions, the post-capture stability check was performed at the center point only. The auxiliary photographs of the lux-meter display used for value recording are retained by the author and are not part of the deposited dataset; the ground-truth values they document are provided in the annotation table and encoded in the image filenames.

### 4.3 Data quality control

Quality control was applied at acquisition time through the center-point stability practice described above, applied both before and after each photograph. This working tolerance corresponds to a few display counts of the meter and is small relative to the meter’s rated accuracy of ±5% of reading over most of the dataset’s illuminance range (for example, 3 lux is 0.5% of the median center-point illuminance of 598 lx, at which ±5% corresponds to ±30 lx). Images failing the check were deleted immediately and re-acquired, so the deposited dataset contains only acquisitions that passed it; the number of repeated acquisitions was not logged, and no post-hoc session exclusions were required. All photographs were acquired during hours of darkness to eliminate daylight and its temporal drift.

### 4.4 Measurement grid and spatial protocol

A grid overlay displayed on the smartphone screen was used to define fixed spatial measurement locations within the image frame. The five-point annotation design included the image center (C) and four peripheral locations: upper left (UL), upper right (UR), lower right (LR), and lower left (LL). For five-point images, illuminance values were recorded at the center and at the four peripheral points. The center point was measured before and after the corner measurements to check illumination stability during the acquisition sequence, as described above. For one-point images, only the central lux measurement was recorded. The grid overlay was used to keep measurement locations spatially consistent across sessions. The five measurement locations correspond to fixed pixel coordinates in the 3060 × 4080 image frame (origin at the top-left corner): C (1520, 2027), UL (1020, 1372), UR (2043, 1372), LR (2030, 2722), LL (1020, 2722). These coordinates are stored as constant columns (x_C … y_LL) in the deposited feature table and are additionally provided as a compact machine-readable file (roi_coordinates.csv). Physical surface coordinates and inter-point distances were not recorded; because the pixel-to-surface mapping depends on camera intrinsics, perspective, and the local surface plane, exact physical coordinates cannot be retrospectively reconstructed, and the deposited native-frame pixel coordinates are the authoritative ROI definitions.

### 4.5 Surface conditions

Images were collected across 1587 measurement sessions, where each session corresponds to one lighting condition (a specific lamp setting and dimmer level). In 1476 sessions, both a bare table surface and a white paper sheet were photographed under the same lighting condition. In 113 of these sessions, up to 22 colored paper sheets were additionally photographed: blush, burgundy, cool red, dark indigo, deep blue, forest green, grass green, lavender, light green, magenta, maroon, midnight blue, navy blue, orange, purple, red, rose, royal blue, steel blue, teal, and yellow. Printed materials were photographed in 38 sessions. In 82 sessions, only white paper was photographed without a corresponding table surface (recorded as Tnone in the session metadata). Ten distinct bare table surface types were used across sessions, representing different materials and colors: beige laminate, brown wood-like surfaces, white tables, and other table types.

Within a colored-paper session, up to 22 sheets were photographed sequentially under a fixed lamp configuration: each sheet was placed at the same marked position, photographed, and replaced by the next sheet without adjusting the lamp. If the lamp position was accidentally disturbed, the session was restarted from the beginning. Sheet placement was repeatable but not pixel-exact: the center measurement point always fell on the paper, while peripheral points could occasionally fall near or beyond the sheet edge; users of five-point annotations on colored paper should treat the center point as the most reliable location.

### 4.6 Lighting conditions

Illumination was generated using two portable dimmable LED lamps, alone or in combination with the fixed luminaires present at the collection locations: (1) a rechargeable cordless LED desk lamp with a 6000 mAh battery, three selectable correlated-color-temperature settings—warm, neutral, and cool—and ten brightness levels; and (2) a PACOVY ADJ-01 adjustable-height gooseneck floor lamp with 120 LED beads, rated power of 18 W, nominal luminous flux of 1800 lm, remote and touch dimming control, and selectable CCT settings of 2700 K, 4000 K, 5000 K, and 6500 K, according to the manufacturer’s documentation. The floor lamp was used at one collection location, a sailboat cabin, either alone or together with the desk lamp. At institutional locations, including a university library, the portable lamps supplemented the existing fixed room lighting, producing mixed-source indoor illumination. Lamp dimming and changes in lamp position were used to generate illuminance values ranging from 1 to 2280 lux across all measured spatial points and to produce different spatial illuminance gradients at the same physical measurement location. Lamp-to-surface distance, incidence angle, and position were deliberately varied and were not recorded as metadata, as acknowledged in the Limitations section. The exact model, rated power, and nominal CCT values of the rechargeable desk lamp were not available. All acquisitions were performed during hours of darkness; therefore, daylight did not contribute to the recorded scenes.

### 4.7 Data collection procedure

Each data collection sequence included the following steps: the lamp color-temperature setting and dimmer level were selected; lux values were recorded for the required annotation design following the measurement and image-capture sequence described above; the photograph was captured with fixed camera settings; and the image filename was assigned to encode the session, table or surface identifier, and lux measurements. For five-point images, the filename includes center and corner lux values. For one-point images, the corner fields are omitted.

### 4.8 Feature extraction

Image features were extracted from fixed regions of interest (ROIs) corresponding to the annotated measurement points. The feature-extraction workflow used a 256 × 256 pixel square ROI centered on the center point and 128 × 128 pixel square ROIs centered on the four peripheral points. Additional features were extracted from a square context region subdivided into a 5 × 5 grid. Extracted features included mean RGB values, mean luma, luma standard deviation, gradient-based features, saturation proxy, ROI coordinate information, square-context features, and spatial-difference features between ROIs. Feature extraction was performed on gamma-encoded 8-bit sRGB values decoded from the deposited JPEG files, without linearization, at the native 3060 × 4080 resolution and after applying EXIF orientation. Luma was computed with Rec. 709 coefficients (Y = 0.2126 R + 0.7152 G + 0.0722 B). Gradient features used NumPy’s central-difference gradient of the luma channel, with gradient magnitude computed as the square root of the sum of squared horizontal and vertical gradients. The saturation proxy is the mean S channel of the HSV conversion (Pillow), scaled to [0, 1]. The derived feature table (feature_master_latest.csv) contains 5814 rows and 133 columns, including metadata, lux labels, ROI coordinates, and image-derived features.

### 4.9 Example analysis workflows

Example machine-learning workflows were implemented to demonstrate reuse of the dataset for lux regression, threshold-based illuminance classification, and color classification under varying illuminance conditions. The example analyses used ExtraTrees models [10] implemented in Python 3.10 with scikit-learn [11]. Grouped train-test splitting was used to reduce potential leakage between related images. Session-based splits assigned all images from the same measurement session to either the training or test set. Physical-point-based splits assigned all images from the same spatial measurement location (physical_point_id) to either set. Grouped splits used scikit-learn GroupShuffleSplit with test_size = 0.2 and random_state = 42; the exact split assignments for the analyses in Fig. 2, Fig. 3, Fig. 4 are deposited as CSV files. XGBoost was also used in the deposited analysis code for comparative regression workflows [12]. Analyses used numpy, pandas, scikit-learn, matplotlib, Pillow, and xgboost; a requirements.txt specifying package versions is provided in the analysis-code repository, and the repository README identifies the scripts and data files generating each figure and table. The complete feature-extraction and analysis code is available at https://doi.org/10.5281/zenodo.20658590 [8].

## Limitations

All images were collected with a single smartphone model (Samsung Galaxy A16) under fixed manual camera settings. The dataset does not represent automatic-exposure photography, other smartphone models, or uncontrolled field conditions; it supports controlled-condition benchmarking rather than field deployment. Some surface categories and illuminance ranges contain fewer images than others; users should verify subset sizes before training or benchmarking models. In early sessions (approximately F001–F059), a paper sheet may not have fully covered all five grid locations, so some corner regions of interest may include the surrounding table surface. Users requiring all regions of interest to lie on a single material should verify coverage using the annotation metadata and image filenames. In a subset of sessions, the lamp was positioned close to one side of the surface to generate high illuminance values, producing pronounced spatial gradients where corner lux values differ substantially from the center measurement. These sessions are intentional variation; spatial uniformity therefore varies across the dataset. Lamp position, lamp-to-surface distance, and incidence angle were varied deliberately but not recorded as metadata, so analyses cannot be stratified by lamp geometry. Physical inter-point distances on the surface were not recorded; the deposited pixel coordinates are the authoritative ROI definitions.

## Ethics Statement

The author has read and followed the ethical requirements for publication in Data in Brief. The current work does not involve human subjects, animal experiments, or data collected from social media platforms. The dataset contains no images of human subjects and no personally identifiable information. All photographs depict inanimate table surfaces and paper sheets under controlled conditions. No ethical approval was required.

## CRediT authorship contribution statement

**Anton Buryi:** Conceptualization, Methodology, Investigation, Data curation, Software, Formal analysis, Visualization, Writing – original draft, Writing – review & editing.

## Data Availability

ZenodoAnalysis Code for Smartphone Image-Based Workplace Illuminance Estimation (Original data)
